# Active Fault Dislocation-Induced Mechanical Response of Polyurethane-Solidified Track in Tunnels

**DOI:** 10.3390/ma18112492

**Published:** 2025-05-26

**Authors:** Wei Chen, Dan Wu, Minzhe Yu, Pei Wu, Yushuo Zhang, Shang Luo, Lei Xu

**Affiliations:** School of Civil Engineering, Central South University, Changsha 410075, China; 224811176@csu.edu.cn (D.W.); 244811165@csu.edu.cn (M.Y.); 18843185690@163.com (P.W.); yushuo.zhang@u.nus.edu (Y.Z.); luoshang1202@163.com (S.L.); leix_2013@163.com (L.X.)

**Keywords:** active fault zone, mechanical response, polyurethane-solidified track bed, track irregularity, dynamic response

## Abstract

The dislocation of the active fault zone altered the stress distribution and geometry of the track structure in the tunnel, which in turn affected the safety and stability of the train operation. Polyurethane-solidified track bed (PSTB) is suitable for sections crossing through active fault zones due to its excellent serviceability and adaptability to deformation. In this study, the stress and deformation response induced by active fault dislocation are investigated for this novel track structure. The corresponding deformation of track structure is subsequently introduced into a vehicle-track dynamics model to calculate the train operation safety index. The study examines the impact of fault displacement on railway track structures, revealing significant vertical deformation in rails that corresponds to the displacement magnitude. The effects are mainly confined to the active fault zone and its immediate surroundings, with variations depending on the fault zone’s structural characteristics. Key factors such as larger displacements, steeper fault angles, and narrower fault zones increase stress on track components, particularly the wide sleeper, which is prone to cracking and represents a structural vulnerability. Higher fault displacement, narrower zones, steeper angles, and increased train speeds elevate derailment risks and wheel load reduction rates, potentially exceeding safety limits. To ensure safety under typical fault conditions, train speeds should not exceed 250 km/h for PSTB with a 40 mm displacement and a 60° fault angle. These findings provide critical guidance for railway construction in fault-prone areas.

## 1. Introduction

With the rapid advance of railway construction into mountainous and seismic-rich areas, it is inevitable that lengthy and extensive active fault zones must be traversed. Influenced by tectonic activities within these fault zones, the tunnel inverted arch and track structure are susceptible to deformation and dislocation along with the surrounding rock, adversely impacting the normal train operation [1]. Consequently, the track structure must possess a certain level of deformation adjustment capability to facilitate rapid repair and restoration to service with minimal engineering measures when deformation occurs in the substructure [2,3]. Polyurethane-solidified track bed (PSTB) represents an elastic consolidated monolithic structure formed by filling the voids between crushed stone ballast with foamed polyurethane material, thereby bonding the ballast particles together [4]. As a novel track structure, PSTB fills the gap between the traditional ballasted track and ballastless track, combining the robust repairability of ballasted track with the high durability of ballastless track [5,6,7].

Currently, researchers have conducted extensive research on the deformation response of tunnel structures crossing active fault zones and the mechanical performance of the PSTB. Cui et al. [8] studied the deformation response and failure mechanism of tunnels subjected to composite fault dislocation and subsequent seismic excitation through numerical simulations. Zhou et al. [9] investigated the deformation and failure mechanism, as well as the lining mechanical response, of flexible-jointed submerged tunnels under strike-slip fault displacement through model tests and numerical simulations. Tian et al. [10] conducted research on seismic fortification zoning for tunnels crossing active fault zones, tunnel deformation and failure mechanisms, seismic fortification measures, and rapid restoration techniques. Zhong et al. [11] established a three-dimensional numerical model for water conveyance tunnels crossing multiple active strike-slip faults, and they proposed two quantitative damage indices. Wang et al. [12] performed a comparative analysis of the structural response patterns and deformation characteristics of tunnels with varying distances from fault zones and under different fault displacement.

The mechanical properties of PSTB tracks under train loading have likewise gained the attention of studying. Xiong et al. [13] studied the influence of PSTB on the natural frequencies of track structures and the vertical and longitudinal vibration transmission characteristics through field measurements. Shao et al. [14] compared the settlements at different points within the transition zone between PSTB and ballastless tracks, examining the effectiveness of polyurethane solidification. Xu et al. [15] designed a PSTB with a wide-slab track structure and investigated its dynamic characteristics and vibration reduction performance. He et al. [16] conducted a comparative analysis of time-domain, frequency-domain, and dynamic indicators between conventional track bed sections and PSTB sections, studying the development characteristics of track irregularities. Xiao et al. [17] monitored the track geometry of a PSTB test section on a high-speed railway for nearly a year after its opening, comparing it with adjacent ballastless track sections as a reference to study and analyzing the quality evolution pattern of PSTB post-commissioning. Cai et al. [18] established a dynamic system model for the vehicle–track–tunnel interaction based on multibody dynamics theory and the finite element method, studying the dynamic characteristics of PSTB in heavy-haul railway tunnels.

While there is abundant research on the damage patterns of tunnels crossing active fault zones and the mechanical properties of PSTB in conventional sections, limited research exists on the mechanical response of PSTB within tunnels under fault dislocation. In detail, fault-induced track deformation is still poorly understood. These dislocation-driven deformations can lead to additional track deformations when trains run through tunnel line segments where faults are present. Its additional effect continues to accumulate until the dynamic specifications of the train operation are exceeded, thus seriously affecting the safety of the train. Therefore, it is necessary to investigate the mapping relationship between fault dislocation and track deformation and the corresponding train dynamics.

In this paper, a numerical model of prefabricated PSTB in tunnels crossing active fault zones is established based on FEM. This model is employed to investigate the fault displacement response of track structures under various fault conditions such as dislocation magnitude, fault zone width and inclination. Based on the deformation results of the rails obtained from simulating fault displacements, a vehicle–track dynamics model developed using MATLAB (2016b) is introduced. The derailment coefficient and wheel load reduction rate are employed as evaluation indicators for vehicle running safety to assess the dynamic response of trains under different displacement fault conditions. This research provides a valuable reference for the construction and development of PSTB in fault zone areas.

## 2. Materials and Methods

### 2.1. Materials and Parameter Determination

Polyurethane is a polymer material formed by the reaction between isocyanate and polyols. Its molecular structure consists of alternating soft segments (polyols) and hard segments (polyisocyanates and chain extenders). After mixing, the polyurethane material infiltrates into the gaps between ballast particles by gravity and pressure. As the reaction proceeds, it foams and expands, forming a material with uniform micro voids that exhibit good bonding properties and elasticity. By filling the pores of the ballast and forming a continuous elastomer with it, polyurethane is an ideal material for solidifying ballast, which helps improve the stability of the track bed and reduce maintenance costs.

**Figure 1:**
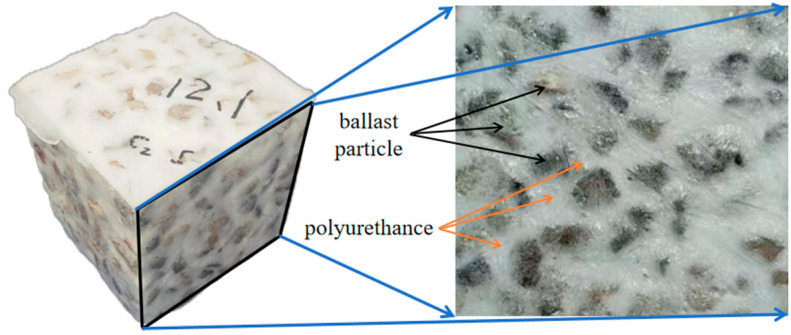
Polyurethane-solidified ballast specimen.

The key point of developing 3D FEM model requires the reasonable constitutive model of polyurethane-solidified ballast. The material constitutive parameter has a significant impact on the simulation results. The uniaxial compression tests can provide an approximate reference for finite element modeling parameters. The isocyanate and polyol prepolymer blend used in the tests was provided by Wanhua Chemical Group (a company in Yantai, China), and the gravel particles were sourced from a building materials factory in Changsha City, China. The specimen preparation steps are as follows: First, the ballast gravel is screened, washed, and dried according to the standard grading requirements for railway ballast to ensure that the cleanliness and grading meet the standards. Then, components A and B are mixed in proportion, with component A mainly being isocyanates and component B containing polyols, chain extenders, and blowing agents. After pasting plastic film on the inner layer of a custom-sized mold, the ballast gravel is poured into it. The uniformly mixed polyurethane material is then evenly poured into the ballast to ensure sufficient bonding between the particles. The polyurethane-solidified ballast specimen obtained after curing and demolding is shown in Figure 1. For details of the experimental procedures and results, please refer to the period literature [19].

The prepared specimens are placed in a servo-loading device for uniaxial loading to monitor the displacement and force during the experiment. Xu et al. [20] conducted uniaxial compression experiments on specimens with ballast gradations of 10–40 mm and 10–35 mm, respectively. The obtained stress–strain curves are approximately linear elastic, with an unconfined compressive stiffness value of approximately 30 MPa. However, since the polyurethane modules in the actual track bed are surrounded by ordinary ballast, the confining pressure exists, leading to the actual elastic modulus value is higher than the unconfined compressive test result. According to the dynamic study results of Cai [18], when the elastic modulus of polyurethane-solidified ballast is lower than 60 MPa, the stress on the rails and sleepers is very large, which will cause large deformations in the track. Therefore, in this paper, the elastic modulus of the polyurethane-solidified ballast module is taken as 60 MPa.

### 2.2. Rock Mass–Tunnel–Track Finite Element Model

**Figure 2:**
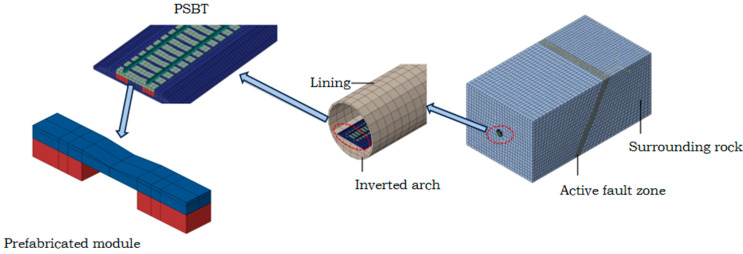
Finite element model of surrounding rock–tunnel–track structure.

A three-dimensional finite element computational model is developed to simulate the interaction dynamics among the surrounding rock, tunnel, and track. This model comprehensively incorporates the footwall rock mass, fault zone, hanging wall rock mass, tunnel structure, and track structure, as illustrated in Figure 2.

The rail, wide sleepers, PSTB modules, ballast and the surrounding rock boundary are all simulated using solid elements, with the mesh type being 8-node element-C3D8R. The meshing process employs a zone-based refinement approach, where sparse meshes are used for parts of the surrounding rock boundary that do not require precise solutions, while dense meshes are applied to the tracks where accuracy is essential. The rationality of this method is demonstrated through the model validation presented in Section 2.6. Surface-to-surface contact interactions are defined between the fault plane and both the hanging wall and the footwall. In this configuration, the hanging wall is fixed, while the footwall is assigned a prescribed displacement. The effect of fault dislocation is simulated by applying displacement boundary conditions to the footwall and fault zone along the dislocation surface, following the initial equilibrium of ground stress. In the PRBT model, surface-to-surface contact is also established between the PRBT blocks and the ballast layer, with the normal contact behavior modeled as “Hard Contact”. Additionally, the wide sleepers and PRBT blocks are rigidly connected using a “Tie” constraint. The Mohr–Coulomb constitutive model is adopted to simulate the elastic–plastic property of the surrounding rock and fault, and the linear elastic constitutive model is used for the tunnel lining.

### 2.3. Vehicle–Track Dynamics Model

Based on the rail deformation results obtained by simulating fault dislocation using the FEM model established in the ABAQUS (2017), a vehicle–track dynamics model developed in MATLAB is presented [21]. In this paper, the irregularities in the track caused by fault dislocation are regarded as supplementary irregularities within the vehicle–track interaction system. By integrating these two sub-models, a co-simulation of track deformation and vehicle–track interaction under fault dislocation conditions is achieved.

**Figure 3:**
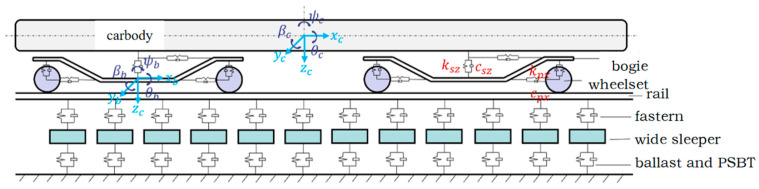
Vehicle-track system.

Within the vehicle–track system, the vehicle subsystem comprises a four-axle vehicle model, encompassing a car body, two bogies, and four wheelsets. Leveraging the finite element formulation of the vehicle–track coupled dynamics model [22], a three-dimensional dynamics model for vehicle–track interaction is formulated, as depicted in Figure 3 [23]. The dynamic equations governing the vehicle–track system are articulated as follows:

(1) $$\left[\begin{array}{cc} M_{VV} & M_{VT}\\ M_{TV} & M_{TT} \end{array}\right]\left\{\begin{array}{c} {\ddot{X}}_{V}\\ {\ddot{X}}_{T} \end{array}\right\}+\left[\begin{array}{cc} C_{VV} & C_{VT}\\ C_{TV} & C_{TT} \end{array}\right]\left\{\begin{array}{c} {\dot{X}}_{V}\\ {\dot{X}}_{T} \end{array}\right\}+\left[\begin{array}{cc} K_{VV} & K_{VT}\\ K_{TV} & K_{TT} \end{array}\right]\left\{\begin{array}{c} X_{V}\\ X_{T} \end{array}\right\}=\left\{\begin{array}{c} F_{V}\\ F_{T} \end{array}\right\}$$

where the subscripts *V* and *T* represent the vehicle system and track system, respectively; *M*, *C*, and *K* represent the mass matrix, damping matrix, and stiffness matrix, respectively; $\ddot{X}$, $\dot{X}$, X represent the acceleration, velocity, and displacement vectors, respectively; and *F* is the load vector.

In the dynamic model, the vehicle has 38 degrees of freedom (DOFs). The rails are modeled as beam elements, with each beam element possessing 12 DOFs. A wide sleeper is represented with 6 DOFs. The vehicle suspension system is configured as a primary suspension, which is assumed to behave as a linear spring-damper system. Rigid contact is adopted for the wheel–rail interaction.

**Figure 4:**
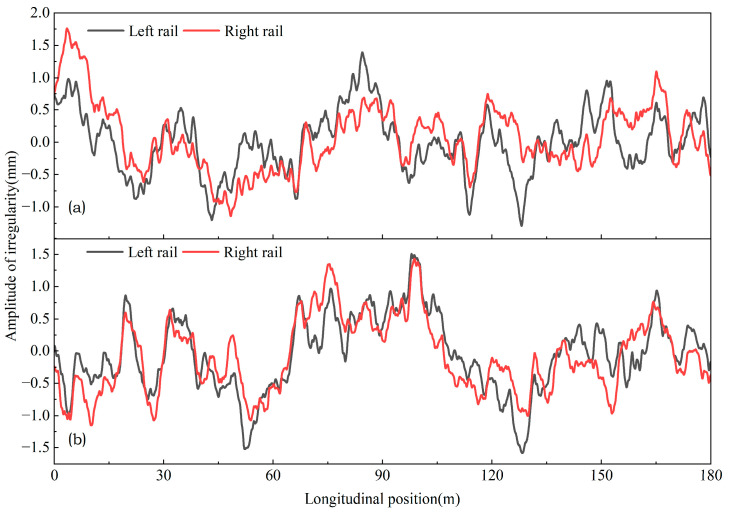
The original track irregularity: (**a**) vertical irregularity; (**b**) lateral irregularity.

During the dynamic calculation process, the irregularity spectrum specific to Chinese high-speed railway ballastless tracks is adopted as the baseline track irregularity, with its spatial distribution illustrated in Figure 4. In subsequent analyses, the deformation of the track resulting from fault dislocation is treated as an additional irregularity superimposed onto the baseline irregularity.

### 2.4. Model Parameters

The dimensions of the rock mass structure are considered as 180 m × 100 m × 80 m to eliminate the influence of boundary conditions, with a width 10.6 times the tunnel diameter and a height 8.5 times the tunnel diameter [24]. The surrounding rock and active fault zone are modeled using the Mohr–Coulomb model, while the lining and inverted arch are modeled using the linear elastic model [25,26,27]. The material parameters are shown in Table 1.

The main components of the prefabricated wide-sleeper polyurethane-solidified track bed include steel rails, wide sleepers, polyurethane-solidified track bed modules, fasteners, and ballast. Notably, steel rails, wide sleepers, prefabricated modules, and ballast are simulated using solid elements with a mesh type of C3D8R [28,29]. The steel rails are CN60 steel rails; the wide sleepers possess a width of 0.4 m, interconnected with the steel rails via fasteners. The nonlinear factors of the fasteners are ignored and simulated using spring-damper elements [30], accounting for stiffness and damping in longitudinal, lateral, and vertical directions, while rotational degrees of freedom of corresponding sleeper nodes are constrained. A separable frictional contact interface is employed between the prefabricated modules and granular ballast [31], whereas a binding constraint is utilized to link the sleepers and prefabricated modules. The material properties of each structural component are outlined in Table 2 [18,32].

### 2.5. Calculation Scheme

The structural attributes of the active fault zone, including fault displacement, width, dip angle, and other factors, will exert varying degrees of influence on the deformation patterns and mechanical responses of the internal track structure. To investigate the differential displacement behavior of the prefabricated wide-sleeper PSTB subject to diverse fault parameters, multiple computational scenarios are established in this study. Specifically, the fault displacement distances considered are 10 mm, 20 mm, 30 mm, and 40 mm; the fault zone widths are 0 m, 5 m, 10 m, 15 m, and 20 m; and fault dip angles of 45°, 60°, and 75° are selected for analysis. The comprehensive scenarios are detailed in Table 3.

### 2.6. Model Validation

**Figure 5:**
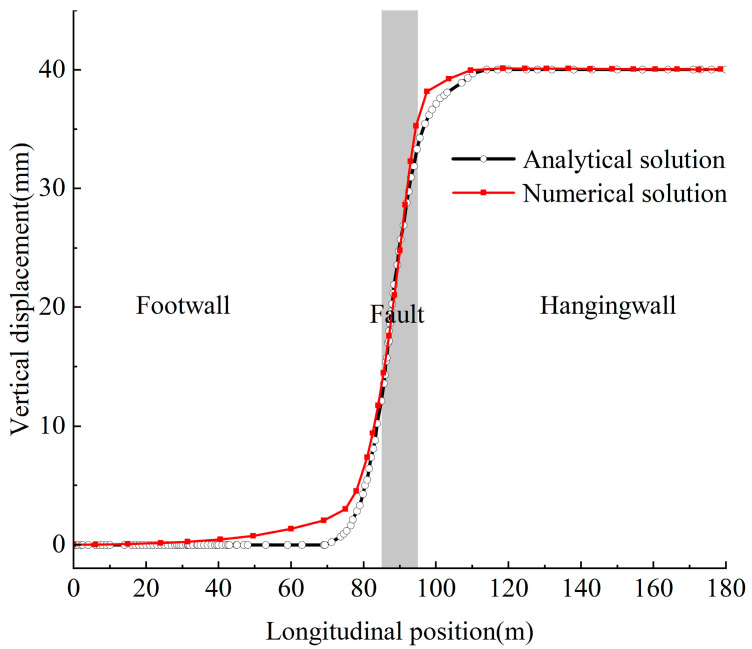
Comparison of numerical solution and analytical solution of vertical displacement of inverted arch.

This paper validates the accuracy of the established model by comparing numerical and analytical solutions for the vertical displacement response of the tunnel inverted arch under normal fault dislocation. The analytical solution is derived from the existing literature [33], whereas the numerical solution stems from the model constructed in this study. As illustrated in Figure 5, a comparison between the numerical and analytical solutions is presented. Minor discrepancies exist between the numerical and analytical results, attributable to the numerical analysis’s utilization of the Mohr–Coulomb constitutive model to simulate the mechanical behavior of the surrounding rock, which the analytical solution is unable to capture, particularly in terms of plastic deformation characteristics. Nevertheless, in terms of trends and numerical ranges, both solutions exhibit good agreement, thereby confirming the correctness of the model presented in this paper. In addition, the vehicle–track dynamics model has been validated in previous work [23]. The numerical results are also in good agreement with the theoretical solution.

## 3. Results and Discussion

### 3.1. Analysis of Track Structure Response Under Normal Fault Dislocation

#### 3.1.1. Impact of Fault Displacement

**Figure 6:**
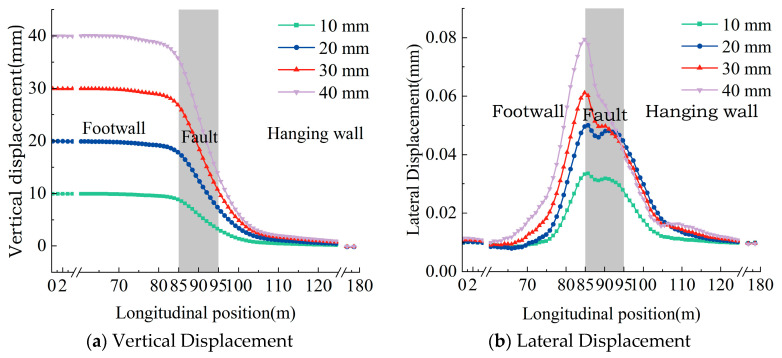
Rail deformation curve under different dislocation displacements.

The vertical and lateral deformation characteristics of the rail under different fault displacements are shown in Figure 6, where the positive vertical displacement indicates relative uplift of the footwall surface. Due to the influence of gravity, fault deformation in the underlying foundation leads to the corresponding deformation in the track structure. Within the fault zone, vertical rail displacement fluctuates significantly along the longitudinal direction, peaking at the boundary with the footwall, approaching the magnitude of the imposed fault displacement. Lateral rail deformation manifests as unilateral bending, with the maximum lateral deformation occurring at the interface between the active fault zone and the footwall. The deformation magnitude escalates with fault displacement; specifically, as fault displacement increases from 10 mm to 40 mm, the maximum lateral deformation increases by factors of 1.49, 1.79, and 2.37, respectively.

**Figure 7:**
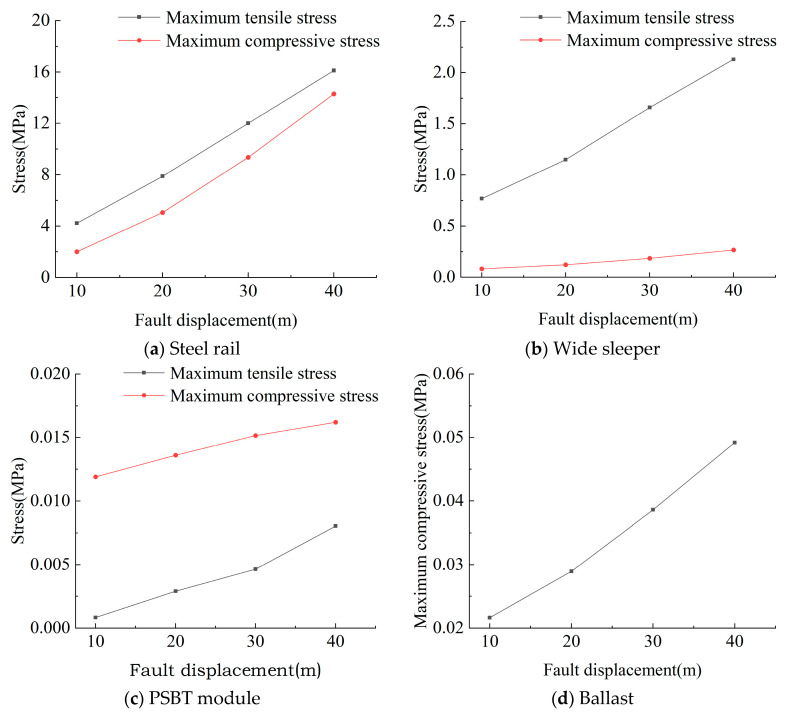
The maximum stress of each component of ballast bed structure under different dislocation displacement.

The computational outcomes of the peak stresses within each component of the track structure across various fault displacements are depicted in Figure 7. As the fault displacement escalates, the maximum tensile and compressive stresses in all track structure components exhibit notable increments. Specifically, as the fault displacement rises from 10 mm to 40 mm, the maximum tensile stress of the rail increases to 1.86 times, 2.84 times, and 3.81 times, respectively, and the maximum compressive stress increases to 2.53 times, 4.78 times, and 7.15 times, respectively; the maximum tensile stress of the wide sleeper increases to 1.49 times, 2.16 times, and 2.77 times, respectively, and the maximum compressive stress increases to 1.48 times, 2.25 times, and 3.25 times, respectively; the maximum tensile stress of the polyurethane-solidified track bed modules increases to 3.45 times, 5.53 times, and 9.96 times, respectively, and the maximum compressive stress increases to 1.14 times, 1.27 times, and 1.36 times, respectively; and the maximum compressive stress of the ballast increases to 1.34 times, 1.78 times, and 2.27 times, respectively.

Among the track structure components, the rail experiences the highest stresses, whereas the polyurethane solidified modules undergo the least. The stress magnitude encountered by each component correlates with its material stiffness; higher stiffness leads to greater stress. The low stiffness and segmented arrangement of the polyurethane solidified modules along the track allow them to remain relatively autonomous during fault movements, thereby experiencing lower stress levels.

#### 3.1.2. Impact of Fault Zone Width

**Figure 8:**
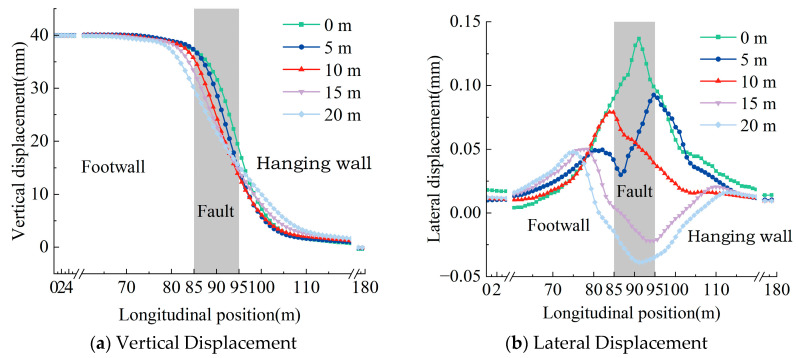
Rail deformation curves under different active fault zone widths.

The deformation of the prefabricated wide sleeper polyurethane-solidified track bed structure under different fault zone widths is shown in Figure 8. For various fault zone widths, the maximum vertical displacement of the rail remains basically consistent with the fault displacement. As the fault zone width increases, the vertical displacement of the rail at the center of the active fault zone decreases, and the range of variation in the “S”-shaped vertical deformation curve of the rail becomes larger. Due to the bending stiffness of the track structure and tunnel, there is a certain amplification effect when the fault displacement influence zone is transmitted towards the internal structure. Therefore, the width of the deformation range of the rail is greater than the width of the fault zone.

When the width of the active fault zone is between 0 m and 10 m, the rail in the prefabricated polyurethane-solidified track bed bends laterally to the left. As the width of the fault zone increases to 15 m and 20 m, the rail bends significantly to the right. With the increase in the width of the active fault zone, the maximum lateral deformation of the rail shows a decreasing trend. When the width of the active fault zone increases from 0 m to 20 m, the maximum lateral deformation decreases to 67.6%, 57.8%, 36.6%, and 35.1%, respectively.

**Figure 9:**
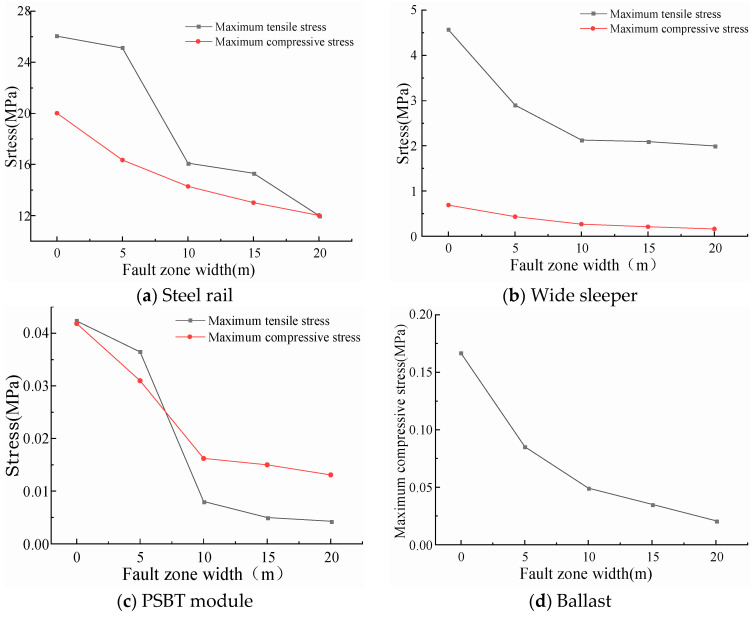
The maximum stress of each component of the ballast bed structure under different active fault zone widths.

**Figure 10:**
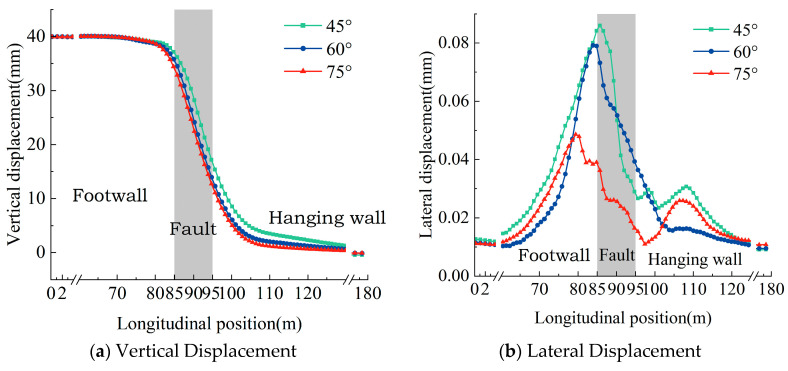
Rail deformation curves under different active fault dip angle.

The results of calculating the maximum stresses in each component of the prefabricated wide sleeper polyurethane solidified track bed, considering that the ballast solely sustains compressive stress, are depicted in Figure 9 as the fault zone width varies from 0 to 20 m. As the width of the active fault zone increases, the maximum tensile and compressive stresses in each component exhibit a decreasing trend. When the fault zone width is less than 10 m, an increase in fault zone width significantly influences the tensile and compressive stresses in each component. Conversely, for fault zone widths exceeding 10 m, the impact of further widening on these stresses gradually diminishes. As illustrated in Figure 10b, at active fault zone widths of 0 m and 5 m, the maximum tensile stresses in the wide sleepers of the prefabricated polyurethane-solidified track bed reach 4.569 MPa and 2.9 Mpa, respectively, surpassing the tensile strength of C60 concrete, which is 2.85 Mpa, leading to damage to the wide sleepers. The wide sleeper, which experiences the second highest stress levels after the rail in the track structure, poses a potential risk of cracking and represents a vulnerable component in the track system levels.

#### 3.1.3. Impact of Fault Zone Dip Angle

Figure 10 presents the vertical and lateral deformation profiles of the prefabricated wide sleeper polyurethane-solidified track bed structure for varying dip angles of the active fault zone. As the fault dip angle augments, the vertical deformation zone within the track structure becomes more localized, accompanied by an escalation in the magnitude of localized deformation. Meanwhile, the lateral deformation amplitude of the rail exhibits a tendency to diminish as the dip angle of the active fault zone increases. Specifically, when the dip angle ascends from 45° to 75°, the maximum lateral deformation of the rail in the prefabricated polyurethane-solidified track bed structure decreases to 91.8% and 56.6% of its original value, respectively.

**Figure 11:**
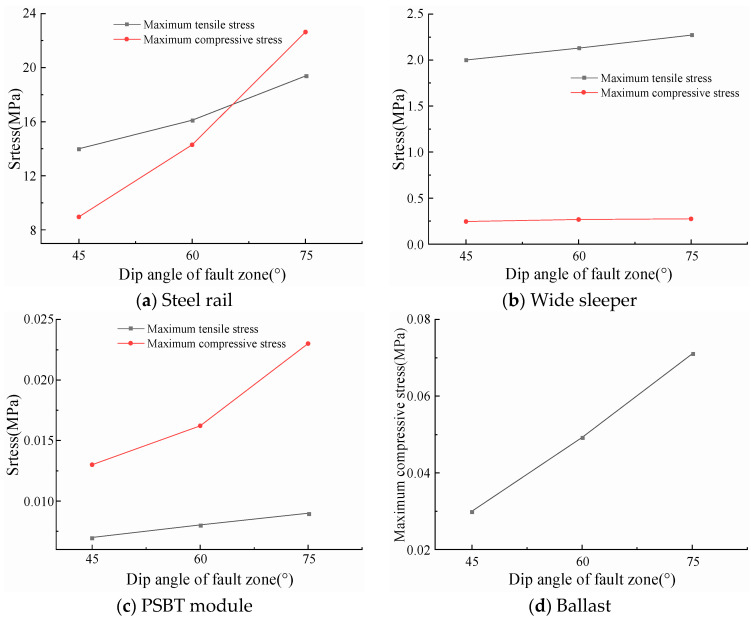
The maximum stress of each component of ballast bed structure under different dip angles of active fault zones.

The calculation results of the maximum stresses in each component of the prefabricated wide sleeper polyurethane-solidified track bed are shown in Figure 11 as the dip angle of the fault zone increases from 45° to 75° at intervals of 15°. With the increase in fault dip angle, the maximum tensile and compressive stresses in each component of the prefabricated wide sleeper polyurethane-solidified track bed show an increasing trend: When the dip angle of the active fault zone increases from 45° to 75°, the maximum tensile stress of the rail increases to 1.15 times and 1.38 times, respectively, and the maximum compressive stress increases to 1.59 times and 2.53 times, respectively; the maximum tensile stress of the wide sleeper increases to 1.06 times and 1.13 times, respectively, and the maximum compressive stress increases to 1.08 times and 1.11 times, respectively; the maximum tensile stress of the polyurethane-solidified track bed module increases to 1.15 times and 1.29 times, respectively, and the maximum compressive stress increases to 1.25 times and 1.77 times, respectively; the maximum compressive stress of the ballast increases to 1.64 times and 2.37 times, respectively. During the process of increasing the dip angle of the active fault zone from 45° to 75°, the maximum stresses In each component of the prefabricated wide sleeper polyurethane-solidified track bed structure do not exceed the strength limits, and the track structure is in a safe state of stress.

### 3.2. Impact of Normal Fault Dislocation on Train Dynamic Response

This paper refers to the Specification for Dynamic Performance Evaluation and Test Appraisal of Rolling Stock (GB/T 5599-2019) [34] to determine the reference limits for the derailment coefficient and wheel load reduction rate. The upper limit of the derailment factor will be set at 0.8. The criteria for assessing the wheel load reduction rate are categorized according to train speed. For train speeds of 160 km/h and below, the upper limit value of wheel load reduction rate is 0.65; for train speeds above 160 km/h, the upper limit value is adjusted to 0.8.

#### 3.2.1. Impact of Fault Displacement

**Figure 12:**
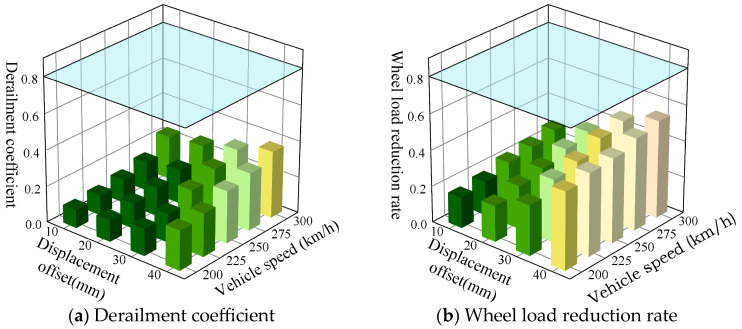
Dynamic response of ballast bed under different dislocation displacement and vehicle speed.

Figure 12 exhibits the peak calculation results for the derailment coefficient and wheel load reduction rate of vehicles traversing the prefabricated wide sleeper polyurethane-solidified track bed at speeds ranging from 200 km/h to 300 km/h, with a fault zone width of 10 m, a dip angle of 60°, and under varying displacement offsets. As both the fault displacement and train speed escalate, there is a corresponding increase in both the derailment coefficient and wheel load reduction rate. Taking the fault displacement of 40 mm as an example, when the train speed increases from 200 km/h to 300 km/h, the derailment coefficient increases to 1.09 times, 1.32 times, 1.45 times, and 1.68 times, respectively, and the wheel load reduction rate increases to 1.07 times, 1.09 times, 1.19 times, and 1.26 times, respectively. Taking the train speed of 300 km/h as an example, when the fault displacement distance increases from 10 mm to 40 mm, the derailment coefficient increases to 1.08 times, 1.29 times, and 1.54 times, respectively, and the wheel load reduction rate increases to 1.21 times, 1.68 times, and 1.93 times, respectively. Within the range of normal fault displacement from 10 mm to 40 mm and train speeds less than 300 km/h, the wheel load reduction rate varies between 0.17 and 0.54, and the derailment coefficient ranges from 0.1 to 0.37. Neither the wheel load reduction rate nor the derailment coefficient exceeds the limit values, meeting the requirements for train operation safety.

#### 3.2.2. Impact of Fault Zone Width

**Figure 13:**
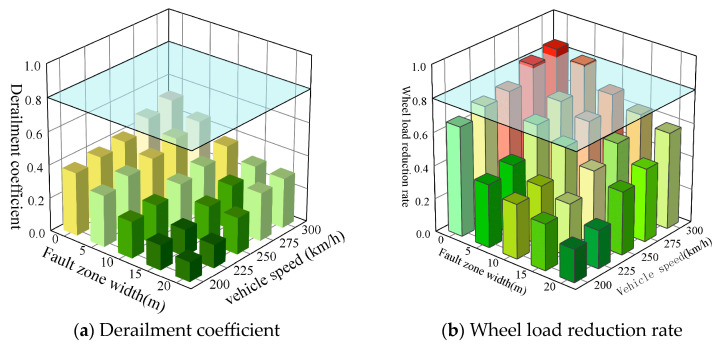
Dynamic response of ballast bed under different fault zone width and vehicle speed.

Figure 13 displays the peak calculation results for the derailment coefficient and wheel load reduction rate of vehicles traveling at speeds ranging from 200 km/h to 300 km/h on the prefabricated wide sleeper polyurethane solidified track bed, with a fault zone displacement of 40 m, a dip angle of 60°, and varying fault zone widths. As the width of the active fault zone decreases and the train speed increases, both the derailment coefficient and wheel load reduction rate exhibit an upward trend. Specifically, in the presence of only an active fault plane, and when the train speeds are 275 km/h and 300 km/h, the wheel load reduction rates reach 0.81 and 0.86, respectively, surpassing the prescribed safety limits. Consequently, under the conditions of a single active fault plane, a displacement of 40 mm, and a fault zone dip angle of 60°, it is recommended to restrict the train speed to 250 km/h to ensure safety.

#### 3.2.3. Impact of Fault Zone Dip Angle

**Figure 14:**
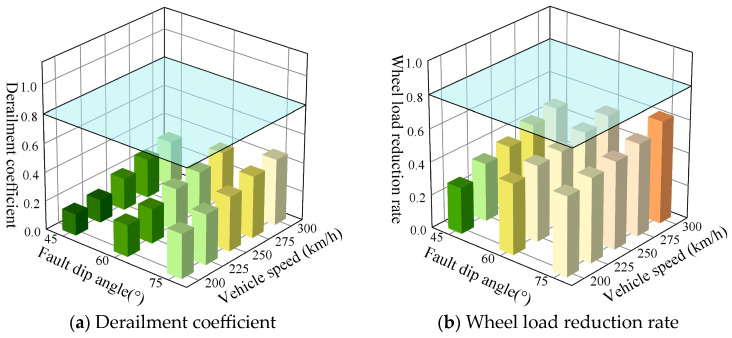
Dynamic response of ballast bed under different fault dip angles and vehicle speeds.

Figure 14 presents the peak calculation results of the derailment coefficient and wheel load reduction rate for vehicles traveling at speeds ranging from 200 km/h to 300 km/h over the prefabricated wide sleeper polyurethane solidified track bed, with the fault zone width of 10 m, the displacement offset of 40 mm, and under different fault zone dip angle conditions. For the prefabricated wide sleeper polyurethane-solidified track bed structure, under conditions of typical fault displacement, it is observed that as both the dip angle of the active fault zone and the train speed escalate, there is a corresponding increase in both the derailment coefficient and wheel load reduction rate. Specifically, when the dip angle of the active fault zone falls within the range of 45° to 75° and the train speed is less than 300 km/h, the wheel load reduction rate fluctuates between 0.28 and 0.62, while the derailment coefficient varies between 0.15 and 0.46. Notably, neither of these parameters exceed their respective limit values, thereby fulfilling the safety requirements for train operations.

## 4. Conclusions

Through numerical simulation, this study focuses on the prefabricated wide sleeper PSTB in tunnels and investigates the response of the track structure in tunnel under the dislocation effects of active fault zones. Initially, a finite element model was constructed to simulate the interactive behavior among the surrounding rock, tunnel, and track structure. By incorporating variables such as dislocation displacement, the width and dip angle of the active fault zone, the deformation and stress distribution patterns of the prefabricated wide sleeper PSTB structure were scrutinized. Furthermore, a vehicle–track coupled dynamic computational model was established to assess the influence of normal fault dislocation on the dynamic responses of trains. Finally, recommendations for safe speed thresholds were subsequently put forward based on evaluation criteria. Key findings are presented below:

1. Under normal fault dislocation, the track structure undergoes substantial vertical deformation, with the rail’s vertical deformation closely approximating the applied displacement value. The deformation range in the rail extends beyond the fault zone’s width. The impact of fault displacement on the deformation and stress state of the track structure primarily concentrates within the active fault zone and its immediate vicinity, with the scope and magnitude of this impact varying based on the structural characteristics of the fault zone and the differing components of the track structure.
2. In the context of normal fault displacement, an increase in the displacement and dip angle of the active fault zone, coupled with a decrease in its width, results in heightened stress levels on each track component.
3. Focused on the PSTB, variations in material properties lead to differing stress experiences among track components. Notably, the wide sleeper, which experiences the second-highest stress levels after the rail in the track structure, poses a potential risk of cracking and represents a structural weakness.
4. An increase in the displacement of the active fault zone, a decrease in its width, an increase in its dip angle, and an increase in train speed all contribute to an elevation in the derailment coefficient and wheel load reduction rate, potentially surpassing safety thresholds to a certain extent. To guarantee train operation safety, under normal fault displacement conditions and for prefabricated wide sleeper polyurethane solidified track beds with only an active fault plane, a displacement of 40 mm, and a fault zone dip angle of 60°, it is advisable to restrict the train speed to 250 km/h.

This study contributes to forecasting the operational safety of trains traveling on PSTB within active fault zones. This study aims to provide the track deformation characteristics and train operation dynamics indicators triggered by fault dislocation features, which can be combined with real-time tunnel deformation detection techniques to predict train operation safety. This study is based on Chinese rail standards, and the applicability to other high-speed railway networks still needs to be explored. In the future, multi-hazard simulations and models for material aging will be carried out in the following aspects: (1) the effect of long-term creep-slip of active fault on the track. (2) the assessment of track safety under sudden dislocation triggered by severe earthquakes.

## Figures and Tables

**Table 1:** Calculation parameters of rock mass and tunnel.

Component	Elastic Modulus (MPa)	Poisson’s Ratio	Density (kg/m^3^)
Surrounding rock	3800	0.31	2200
Fault zone	500	0.45	1900
Lining	32,500	0.2	2400

**Table 2:** Parameter table of track structure material for prefabricated PSTB.

Component	Parameters	Unit	Value
Steel rail	Density	kg/m^3^	7850
	Elastic modulus	MPa	2.6 × 10^5^
	Poisson’s ratio	/	0.167
Wide sleeper	Density	kg/m^3^	2500
	Elastic modulus	MPa	3.65 × 10^4^
	Poisson’s ratio	/	0.2
Polyurethane solidified track bed	Density	kg/m^3^	1900
	Elastic modulus	MPa	60
	Poisson’s ratio	/	0.17
	α damping coefficient	/	3
	β damping coefficient	/	2.5 × 10^−5^
Ballast	Density	kg/m^3^	1800
	Elastic modulus	MPa	130
	Poisson’s ratio	/	0.27
	α damping coefficient	/	3
	β damping coefficient	/	2.5 × 10^−5^
Fastener system	Vertical stiffness	MN/m	120
	Lateral stiffness	MN/m	40
	Longitudinal stiffness	MN/m	20
	Vertical damping	N·s/m	2 × 10^4^
	Lateral damping	N·s/m	2 × 10^4^
	Longitudinal damping	N·s/m	2 × 10^4^
	Vertical stiffness	MN/m	120

**Table 3:** Calculated work conditions.

Work Condition	Displacement Due to Fault Movement (mm)	Width of Fault Zone (m)	Dip Angle of Fault Zone (°)
1–4	10/20/30/40	10	60
5–9	40	0/5/10/15/20	60
10–12	40	10	45/60/75

## Data Availability

The original contributions presented in this study are included in the article. Further inquiries can be directed to the corresponding author.
